# 3-Benzoyl-5-chloro­uracil

**DOI:** 10.1107/S1600536809001287

**Published:** 2009-01-17

**Authors:** Graeme J. Gainsford, Keith Clinch

**Affiliations:** a Industrial Research Limited, PO Box 31-310, Lower Hutt, New Zealand

## Abstract

The dihedral angle between the planes of two aromatic rings in the title compound [systematic name: 3-benzoyl-5-chloro-pyrimidine-2,4(1*H*,3*H*)-dione], C_11_H_7_ClN_2_O_3_, is 86.79 (6)°. Centrosymmetric dimers formed by N—H⋯O hydrogen bonds are linked through C—H⋯O inter­actions, forming a two-dimensional network parallel to (10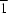).

## Related literature

For a related structure, see: Parvez *et al.* (2007 ▶). For graph-set notation, see: Bernstein *et al.* (1995 ▶). For the synthesis, see: Birck *et al.* (2009 ▶).
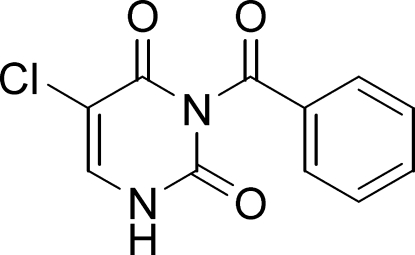


## Experimental

### 

#### Crystal data

C_11_H_7_ClN_2_O_3_
*M* *_r_* = 250.64
Monoclinic, 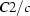

*a* = 21.9357 (9) Å
*b* = 5.4020 (2) Å
*c* = 19.9642 (9) Å
β = 113.169 (2)°
*V* = 2174.89 (16) Å^3^
*Z* = 8
Mo *K*α radiation
μ = 0.35 mm^−1^
*T* = 133 (2) K
0.34 × 0.21 × 0.03 mm

#### Data collection

Bruker APEXII CCD area-detector diffractometer
Absorption correction: multi-scan (*SADABS*; Blessing, 1995 ▶; Bruker, 2006 ▶) *T*_min_ = 0.810, *T*_max_ = 0.990
24616 measured reflections
2899 independent reflections
2189 reflections with *I* > 2σ(*I*)
*R*_int_ = 0.047

#### Refinement

*R*[*F* ^2^ > 2σ(*F* ^2^)] = 0.041
*wR*(*F* ^2^) = 0.110
*S* = 1.07
2899 reflections
162 parameters
H atoms treated by a mixture of independent and constrained refinement
Δρ_max_ = 0.37 e Å^−3^
Δρ_min_ = −0.42 e Å^−3^

### 

Data collection: *APEX2* (Bruker, 2006 ▶); cell refinement: *SAINT* (Bruker, 2006 ▶); data reduction: *SAINT*; program(s) used to solve structure: *SHELXS97* (Sheldrick, 2008 ▶); program(s) used to refine structure: *SHELXL97* (Sheldrick, 2008 ▶); molecular graphics: *ORTEP* in *WinGX* (Farrugia, 1999 ▶) and *Mercury* (Bruno *et al.*, 2002 ▶); software used to prepare material for publication: *SHELXL97* and *PLATON* (Spek, 2003 ▶).

## Supplementary Material

Crystal structure: contains datablocks global, I. DOI: 10.1107/S1600536809001287/ci2757sup1.cif

Structure factors: contains datablocks I. DOI: 10.1107/S1600536809001287/ci2757Isup2.hkl

Additional supplementary materials:  crystallographic information; 3D view; checkCIF report

## Figures and Tables

**Table 1:** Hydrogen-bond geometry (Å, °)

*D*—H⋯*A*	*D*—H	H⋯*A*	*D*⋯*A*	*D*—H⋯*A*
N1—H1*N*⋯O14^i^	0.86 (3)	1.91 (3)	2.770 (2)	173 (3)
C9—H9⋯O15^ii^	0.95	2.46	3.182 (3)	133
C10—H10⋯O15^iii^	0.95	2.57	3.447 (3)	153

Symmetry codes: (i) 
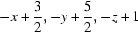
; (ii) 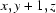; (iii) 
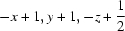
.

**Fig. 1.**
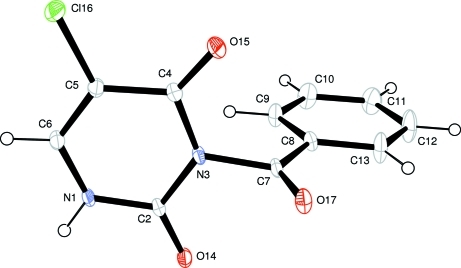
Molecular structure of the title compound. Displacement ellipsoids are shown at the 30% probability level.

**Fig. 2.**
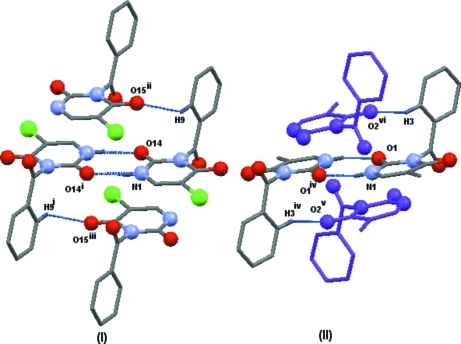
Comparison of similar hydrogen bond interactions (dotted lines) in (I) & (II) (MERCURY; Bruno et al., 2002). Only the hydrogen atoms involved are included. Nitrogen and Oxygen atoms are shown as balls. Colours: Nitrogen, blue (light gray); Oxygen, red (black); Carbon, (gray); Hydrogen, dark blue. Coordinates of (II) are labelled as in deposited data (SEVQUG) and additional molecules shown in purple for clarity. Symmetry Codes: (i) 3/2 - x, 5/2 - y, 1 - z (ii) x, y + 1, z (iii) 3/2 - x, 3/2 - y, 1 - z (iv) 2 - x, 1 - y, -z (v) 1/2 + x, 1/2 - y, -z (vi) 3/2 - x, 1/2 + y, z

## supplementary crystallographic information

### Comment

The title compound, (I), was prepared for incorporation into potential thymidine phosphorylase inhibitors (Birck *et al.*, 2009). Its molecular structure is shown in Fig.1, labelled in the same way as the closely related 5-methyl adduct, 3-benzoylthymine (II) (Parvez *et al.*, 2007). The dihedral angle between the aromatic rings in (I) is 86.79 (10)° compared with 83.82 (6)° in (II). The N3–C7–C8–C9 torsion angle of the ring-linkage is -6.9 (2)° in (I) and -11.8 (2)° in (II). Bond distances are normal.

The crystal packing is dominated by centrosymmetric N1—H1N···O14 hydrogen bonded dimers (common graph-set *R*_2_^2^(8), Bernstein *et al.*, 1995) linked by weaker C–H···O interactions (Table 1). These two types of packing interactions are also found in (II), though not reported, as is illustrated in the comparison Fig 2. The replacement of the methyl group in (II) by chlorine in (I) has not enhanced the packing interactions: neither group/atom play a significant role.

### Experimental

Synthetic details are given in Birck *et al.* (2009).

### Refinement

Atoms H1N and H6 were located in a difference map and refined freely. All other H atoms were restrained using riding models (C-H = 0.95 Å), with *U*_iso_ values constrained to 1.2 times that of the *U*_eq_ of their parent atom.

### Crystal data

**Table:**

C_11_H_7_ClN_2_O_3_	*F*(000) = 1024
*M**_r_* = 250.64	*D*_x_ = 1.531 Mg m^−^^3^
Monoclinic, *C*2/*c*	Mo *K*α radiation, λ = 0.71073 Å
Hall symbol: -C 2yc	Cell parameters from 6298 reflections
*a* = 21.9357 (9) Å	θ = 2.2–28.7°
*b* = 5.4020 (2) Å	µ = 0.35 mm^−^^1^
*c* = 19.9642 (9) Å	*T* = 133 K
β = 113.169 (2)°	Plate, colourless
*V* = 2174.89 (16) Å^3^	0.34 × 0.21 × 0.03 mm
*Z* = 8	

### Data collection

**Table:**

Bruker–Nonius APEXII CCD area-detector diffractometer	2899 independent reflections
Radiation source: fine-focus sealed tube	2189 reflections with *I* > 2σ(*I*)
graphite	*R*_int_ = 0.047
Detector resolution: 8.333 pixels mm^-1^	θ_max_ = 29.0°, θ_min_ = 3.5°
φ and ω scans	*h* = −29→29
Absorption correction: multi-scan (*SADABS*; Blessing, 1995; Bruker, 2006)	*k* = −7→7
*T*_min_ = 0.810, *T*_max_ = 0.990	*l* = −27→27
24616 measured reflections	

### Refinement

**Table:**

Refinement on *F*^2^	Primary atom site location: structure-invariant direct methods
Least-squares matrix: full	Secondary atom site location: difference Fourier map
*R*[*F*^2^ > 2σ(*F*^2^)] = 0.041	Hydrogen site location: inferred from neighbouring sites
*wR*(*F*^2^) = 0.110	H atoms treated by a mixture of independent and constrained refinement
*S* = 1.07	*w* = 1/[σ^2^(*F*_o_^2^) + (0.0468*P*)^2^ + 2.9413*P*] where *P* = (*F*_o_^2^ + 2*F*_c_^2^)/3
2899 reflections	(Δ/σ)_max_ = 0.001
162 parameters	Δρ_max_ = 0.37 e Å^−^^3^
0 restraints	Δρ_min_ = −0.42 e Å^−^^3^

### Special details

**Table:**

Geometry. All e.s.d.'s (except the e.s.d. in the dihedral angle between two l.s. planes) are estimated using the full covariance matrix. The cell e.s.d.'s are taken into account individually in the estimation of e.s.d.'s in distances, angles and torsion angles; correlations between e.s.d.'s in cell parameters are only used when they are defined by crystal symmetry. An approximate (isotropic) treatment of cell e.s.d.'s is used for estimating e.s.d.'s involving l.s. planes.
Refinement. An extinction parameter was refined. Refinement of *F*^2^ against ALL reflections. The weighted *R*-factor *wR* and goodness of fit *S* are based on *F*^2^, conventional *R*-factors *R* are based on *F*, with *F* set to zero for negative *F*^2^. The threshold expression of *F*^2^ > σ(*F*^2^) is used only for calculating *R*-factors(gt) *etc*. and is not relevant to the choice of reflections for refinement. *R*-factors based on *F*^2^ are statistically about twice as large as those based on *F*, and *R*- factors based on ALL data will be even larger.

### Fractional atomic coordinates and isotropic or equivalent isotropic displacement parameters (Å^2^)

**Table:**

	*x*	*y*	*z*	*U*_iso_*/*U*_eq_	
Cl16	0.72366 (2)	0.47956 (9)	0.28923 (3)	0.03151 (14)	
O14	0.68050 (6)	1.0941 (2)	0.50072 (7)	0.0230 (3)	
O15	0.61273 (7)	0.4522 (3)	0.34170 (8)	0.0316 (3)	
O17	0.60738 (6)	0.5865 (3)	0.49746 (7)	0.0259 (3)	
N1	0.73929 (7)	1.0080 (3)	0.43155 (8)	0.0196 (3)	
H1N	0.7670 (11)	1.123 (5)	0.4545 (12)	0.034 (6)*	
N3	0.64590 (6)	0.7803 (3)	0.41885 (8)	0.0185 (3)	
C2	0.68836 (8)	0.9710 (3)	0.45337 (9)	0.0178 (3)	
C4	0.65184 (8)	0.6191 (3)	0.36658 (10)	0.0212 (4)	
C5	0.70867 (8)	0.6748 (3)	0.34898 (10)	0.0206 (3)	
C6	0.74965 (8)	0.8616 (3)	0.38121 (10)	0.0204 (3)	
H6	0.7865 (11)	0.905 (4)	0.3704 (11)	0.025 (5)*	
C7	0.59307 (8)	0.7215 (3)	0.44632 (10)	0.0189 (3)	
C8	0.52873 (8)	0.8413 (3)	0.40703 (10)	0.0204 (4)	
C9	0.51969 (9)	1.0174 (4)	0.35368 (11)	0.0280 (4)	
H9	0.5554	1.0604	0.3403	0.034*	
C10	0.45871 (10)	1.1310 (4)	0.31977 (12)	0.0367 (5)	
H10	0.4524	1.2524	0.2832	0.044*	
C11	0.40709 (10)	1.0666 (5)	0.33943 (13)	0.0398 (5)	
H11	0.3650	1.1426	0.3156	0.048*	
C12	0.41587 (10)	0.8944 (5)	0.39288 (14)	0.0458 (6)	
H12	0.3800	0.8527	0.4061	0.055*	
C13	0.47664 (10)	0.7816 (4)	0.42752 (13)	0.0375 (5)	
H13	0.4830	0.6640	0.4651	0.045*	

### Atomic displacement parameters (Å^2^)

**Table:**

	*U*^11^	*U*^22^	*U*^33^	*U*^12^	*U*^13^	*U*^23^
Cl16	0.0351 (3)	0.0321 (3)	0.0358 (3)	−0.0100 (2)	0.0230 (2)	−0.0132 (2)
O14	0.0189 (6)	0.0250 (6)	0.0294 (7)	−0.0055 (5)	0.0141 (5)	−0.0060 (5)
O15	0.0311 (7)	0.0315 (8)	0.0372 (8)	−0.0164 (6)	0.0186 (6)	−0.0120 (6)
O17	0.0181 (6)	0.0285 (7)	0.0323 (7)	0.0009 (5)	0.0112 (5)	0.0095 (6)
N1	0.0141 (6)	0.0217 (7)	0.0250 (8)	−0.0068 (6)	0.0098 (6)	−0.0051 (6)
N3	0.0131 (6)	0.0190 (7)	0.0249 (8)	−0.0042 (5)	0.0091 (6)	−0.0012 (6)
C2	0.0122 (7)	0.0185 (8)	0.0225 (8)	−0.0023 (6)	0.0065 (6)	0.0010 (6)
C4	0.0189 (8)	0.0226 (8)	0.0230 (9)	−0.0047 (7)	0.0091 (7)	−0.0011 (7)
C5	0.0193 (8)	0.0212 (8)	0.0239 (9)	−0.0016 (6)	0.0112 (7)	−0.0022 (7)
C6	0.0157 (7)	0.0232 (8)	0.0236 (9)	−0.0014 (7)	0.0092 (7)	−0.0001 (7)
C7	0.0138 (7)	0.0194 (8)	0.0257 (9)	−0.0047 (6)	0.0099 (7)	−0.0015 (7)
C8	0.0143 (7)	0.0212 (8)	0.0259 (9)	−0.0010 (6)	0.0079 (7)	0.0027 (7)
C9	0.0210 (8)	0.0324 (10)	0.0341 (10)	0.0011 (8)	0.0145 (8)	0.0095 (8)
C10	0.0299 (10)	0.0415 (12)	0.0403 (13)	0.0105 (9)	0.0156 (9)	0.0181 (10)
C11	0.0218 (9)	0.0514 (14)	0.0462 (13)	0.0136 (9)	0.0136 (9)	0.0153 (11)
C12	0.0188 (9)	0.0631 (16)	0.0621 (16)	0.0099 (10)	0.0229 (10)	0.0265 (13)
C13	0.0210 (9)	0.0467 (13)	0.0504 (13)	0.0052 (9)	0.0202 (9)	0.0245 (10)

### Geometric parameters (Å, °)

**Table:**

Cl16—C5	1.7179 (18)	C7—C8	1.468 (2)
O14—C2	1.222 (2)	C8—C9	1.383 (3)
O15—C4	1.209 (2)	C8—C13	1.394 (2)
O17—C7	1.192 (2)	C9—C10	1.382 (3)
N1—C2	1.364 (2)	C9—H9	0.95
N1—C6	1.366 (2)	C10—C11	1.381 (3)
N1—H1N	0.87 (3)	C10—H10	0.95
N3—C2	1.378 (2)	C11—C12	1.371 (3)
N3—C4	1.404 (2)	C11—H11	0.95
N3—C7	1.498 (2)	C12—C13	1.379 (3)
C4—C5	1.453 (2)	C12—H12	0.95
C5—C6	1.336 (2)	C13—H13	0.95
C6—H6	0.94 (2)		
			
C2—N1—C6	122.97 (15)	C8—C7—N3	115.49 (14)
C2—N1—H1N	115.5 (15)	C9—C8—C13	119.96 (17)
C6—N1—H1N	121.3 (15)	C9—C8—C7	122.09 (15)
C2—N3—C4	126.39 (14)	C13—C8—C7	117.87 (16)
C2—N3—C7	116.31 (14)	C10—C9—C8	120.02 (17)
C4—N3—C7	116.87 (13)	C10—C9—H9	120.0
O14—C2—N1	123.34 (15)	C8—C9—H9	120.0
O14—C2—N3	121.36 (14)	C11—C10—C9	119.52 (19)
N1—C2—N3	115.29 (15)	C11—C10—H10	120.2
O15—C4—N3	120.65 (15)	C9—C10—H10	120.2
O15—C4—C5	126.35 (17)	C12—C11—C10	120.82 (19)
N3—C4—C5	113.00 (15)	C12—C11—H11	119.6
C6—C5—C4	121.21 (16)	C10—C11—H11	119.6
C6—C5—Cl16	121.45 (13)	C11—C12—C13	120.15 (19)
C4—C5—Cl16	117.23 (13)	C11—C12—H12	119.9
C5—C6—N1	121.09 (16)	C13—C12—H12	119.9
C5—C6—H6	123.4 (13)	C12—C13—C8	119.51 (19)
N1—C6—H6	115.4 (13)	C12—C13—H13	120.2
O17—C7—C8	126.97 (15)	C8—C13—H13	120.2
O17—C7—N3	117.53 (15)		
			
C6—N1—C2—O14	176.60 (17)	C2—N3—C7—O17	−84.6 (2)
C6—N1—C2—N3	−2.5 (2)	C4—N3—C7—O17	88.4 (2)
C4—N3—C2—O14	−176.59 (17)	C2—N3—C7—C8	94.63 (18)
C7—N3—C2—O14	−4.3 (2)	C4—N3—C7—C8	−92.32 (19)
C4—N3—C2—N1	2.6 (2)	O17—C7—C8—C9	172.27 (19)
C7—N3—C2—N1	174.86 (14)	N3—C7—C8—C9	−6.9 (3)
C2—N3—C4—O15	177.38 (17)	O17—C7—C8—C13	−4.6 (3)
C7—N3—C4—O15	5.1 (3)	N3—C7—C8—C13	176.25 (18)
C2—N3—C4—C5	−1.5 (2)	C13—C8—C9—C10	−1.2 (3)
C7—N3—C4—C5	−173.75 (15)	C7—C8—C9—C10	−177.94 (19)
O15—C4—C5—C6	−178.47 (19)	C8—C9—C10—C11	−0.2 (4)
N3—C4—C5—C6	0.3 (3)	C9—C10—C11—C12	1.0 (4)
O15—C4—C5—Cl16	−2.2 (3)	C10—C11—C12—C13	−0.4 (4)
N3—C4—C5—Cl16	176.58 (12)	C11—C12—C13—C8	−0.9 (4)
C4—C5—C6—N1	−0.4 (3)	C9—C8—C13—C12	1.7 (4)
Cl16—C5—C6—N1	−176.52 (14)	C7—C8—C13—C12	178.6 (2)
C2—N1—C6—C5	1.6 (3)		

### Hydrogen-bond geometry (Å, °)

**Table:**

*D*—H···*A*	*D*—H	H···*A*	*D*···*A*	*D*—H···*A*
N1—H1N···O14^i^	0.86 (3)	1.91 (3)	2.770 (2)	173 (3)
C9—H9···O15^ii^	0.95	2.46	3.182 (3)	133
C10—H10···O15^iii^	0.95	2.57	3.447 (3)	153

Symmetry codes: (i) −*x*+3/2, −*y*+5/2, −*z*+1; (ii) *x*, *y*+1, *z*; (iii) −*x*+1, *y*+1, −*z*+1/2.

## References

[bb1] Bernstein, J., Davis, R. E., Shimoni, L. & Chang, N.-L. (1995). *Angew. Chem. Int. Ed. Engl.* **34**, 1555–1573.

[bb2] Birck, M. T., Clinch, K., Gainsford, G. J., Schramm, V. L. & Tyler, P. C. (2009). *Helv. Chim. Acta.* Submitted.

[bb3] Blessing, R. H. (1995). *Acta Cryst.* A**51**, 33–38. 10.1107/s0108767394005726 7702794

[bb4] Bruker (2006). *APEX2*, *SAINT* and *SADABS* Bruker AXS Inc., Madison, Wisconsin, USA.

[bb5] Bruno, I. J., Cole, J. C., Edgington, P. R., Kessler, M., Macrae, C. F., McCabe, P., Pearson, J. & Taylor, R. (2002). *Acta Cryst.* B**58**, 389–397. 10.1107/s0108768102003324 12037360

[bb6] Farrugia, L. J. (1999). *J. Appl. Cryst.* **32**, 837–838.

[bb7] Parvez, M., Phillips, S. E. & Sutherland, T. C. (2007). *Acta Cryst.* E**63**, o733–o734.

[bb8] Sheldrick, G. M. (2008). *Acta Cryst.* A**64**, 112–122. 10.1107/S0108767307043930 18156677

[bb9] Spek, A. L. (2003). *J. Appl. Cryst.* **36**, 7–13.

